# ﻿*Rhynchospioaciliata* sp. nov., a new spionid species (Annelida, Spionidae) from the Korea Strait

**DOI:** 10.3897/zookeys.1100.80077

**Published:** 2022-05-16

**Authors:** Geon Hyeok Lee, Gi-Sik Min

**Affiliations:** 1 Department of Biological Sciences and Bioengineering, Inha University, Incheon 22212, Republic of Korea Inha University Incheon Republic of Korea

**Keywords:** Korean waters, methyl green staining pattern, molecular analysis, morphology, taxonomy

## Abstract

A new spionid polychaete, *Rhynchospioaciliata***sp. nov.**, was discovered in the fine sandy sediments of an intertidal habitat from Korean waters. The new species is considered a simultaneous hermaphrodite, but no brooding embryos were found in any of the specimens collected in this study. This species is unique in the absence of ciliation in the anteriormost chaetigers. *Rhynchospioaciliata***sp. nov.** is morphologically most similar to *Rhynchospiofoliosa* Imajima, 1991 from Japan in having an elevation on the prostomium, conspicuously large and foliaceous branchiae, and intersegmental lateral pouches. However, the new species differs from the latter by the following characteristics: (1) large and lanceolate notopodial postchaetal lamellae of chaetiger 1, (2) transverse ciliated bands and ciliation on the inner branchiae absent in anteriormost chaetigers, and (3) pygidium with one pair of ventral cirri and numerous elongated dorsolateral cirri. Detailed description and illustrations of the new species are provided with molecular information on mitochondrial cytochrome c oxidase subunit I (COI), 16S ribosomal DNA (rDNA), nuclear 18S rDNA, and 28S rDNA.

## ﻿Introduction

*Rhynchospio* Hartman, 1936 is one of the less species-rich spionid genera mainly reported from the Pacific and adjacent waters and comprising 12 valid species (Radashevsky and Choi 2021). Some *Rhynchospio* species are known to be simultaneous hermaphrodites and brood their larvae on the dorsum (Radashevsky 2007; Radashevsky and Choi 2021). Adult members of the genus are characterized by a prostomium with frontolateral horns, branchiae appearing from chaetiger 2, notopodia with only capillary chaetae, and more than two pairs of pygidial cirri (Fauchald 1977; Blake and Kudenov 1978; Radashevsky et al. 2016). Some morphological characteristics, such as the first appearance of neuropodial hooded hooks and the number of dorsal pygidium cirri are highly variable and overlap between *Rhynchospio* species (Blake 1996; Radashevsky et al. 2014). For this reason, the status of some *Rhynchospio* species remains uncertain (e.g., R.cf.foliosa from USA, see Radashevsky et al. 2016). Identifying *Rhynchospio* species using only morphological characters has been considered very difficult, and a combination of detailed morphological and molecular analyses is required (Radashevsky et al. 2014; Simon et al. 2018). Additionally, information from the methyl green staining pattern (MGSP) can also be important for identifying *Rhynchospio* species (Simon et al. 2018).

In northeast Asia, four *Rhynchospio* species, *R.asiatica* Chlebovitsch, 1959; *R.foliosa Imajima*, 1991; *R.glandulosa Radashevsky* & Choi, 2021 and *R.tuberculata* Imajima, 1991, have been recorded (Chlebovitsch 1959; Imajima 1991; Radashevsky et al. 2014; Abe and Sato-Okoshi 2021; Radashevsky and Choi 2021). One *Rhynchospio* species, *R.glandulosa*, has been reported from Korean waters (Radashevsky and Choi 2021). In this study, a new *Rhynchospio*, *R.aciliata* sp. nov. was discovered in the intertidal sandflats of the Korea Strait. Detailed description and illustrations of the new species are provided together with partial DNA sequences of four gene regions (mitochondrial cytochrome c oxidase subunit I [COI], 16S ribosomal DNA [rDNA], nuclear 18S rDNA, and 28S rDNA).

## ﻿Materials and methods

### ﻿Sampling and morphological observations

**Figure 1.**
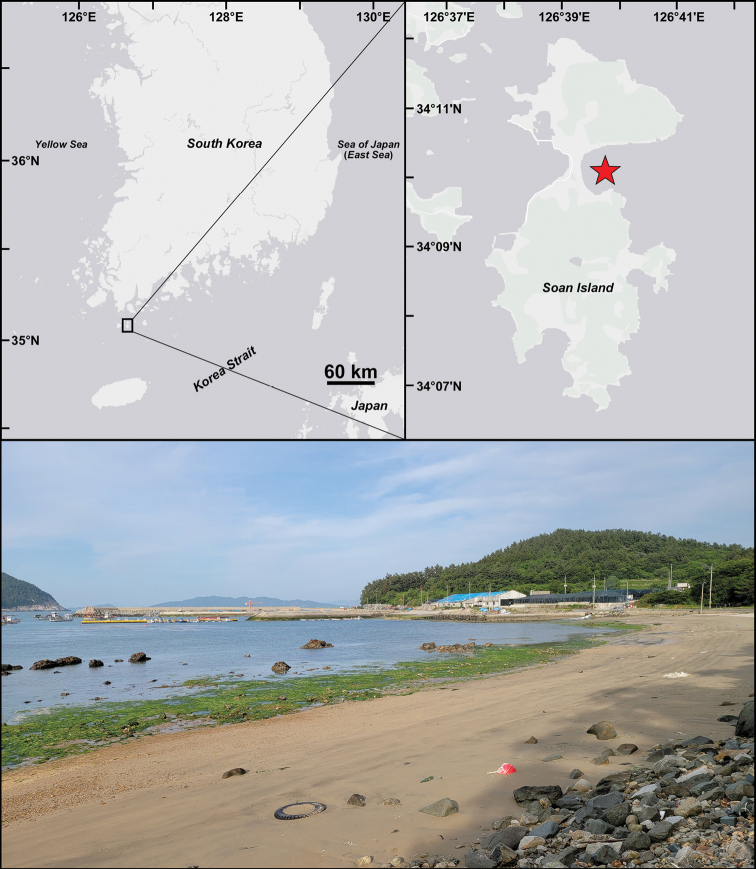
Map and habitat of the type locality (red star) where *Rhynchospioaciliata* sp. nov. were collected.

Adult samples were collected from sandflats in the intertidal zone of the Korea Strait (Fig. 1) using 500 μm mesh sieves. Morphological observations were performed on both live and formalin-fixed materials. The live specimens were relaxed using 10% MgCl2 solution in seawater, and characteristics were observed under a stereomicroscope (Leica MZ125, Microsystems Wetzlar GmbH, Wetzlar, Germany). After live observation, the individuals were fixed in 4% formaldehyde for morphological study and subsequently transferred to 70% ethanol. Some formalin-fixed specimens (intermittently transferred to distilled water) were stained with methyl green solution according to the method described by Meißner (2005). To observe the morphology of sperm and oocytes, a few formalin-fixed specimens were transferred to glycerol. Photographs were taken using a digital camera (Tucsen Dhyana 400DC, Fuzhou Fujian, China) with the capture program Tucsen Mosaic v. 15 (Fuzhou Fujian, China). Dissected appendages were mounted using the Eukitt Quick-hardening mounting medium (Sigma-Aldrich, St. Louis, MO, USA) for permanent slides. The specimens were fixed in 95% ethanol for molecular analyses. The specimens for scanning electron microscopy (SEM) were dehydrated using a t-BuOH freeze dryer (VFD-21S Vacuum Device; Ibaraki, Japan), covered with platinum, and observed using a Hitachi SEM model S-4300SE (Hitachi, Japan). All type and voucher specimens examined in this study were deposited at the National Institute of Biological Resources at Incheon, South Korea (NIBR). Non-type material of *R.foliosa* from Akkeshi, Hokkaido, northern Japan (NSMT-Pol 104819) deposited in National Museum of Nature and Science was also examined for morphological comparison.

### ﻿Molecular analysis

Genomic DNA was extracted from the palps of three specimens of the new species (NIBRIV0000893846–8) using a LaboPass Tissue Mini (Cosmo GENETECH, Seoul, South Korea) according to the manufacturer’s instructions. PCR amplification of mitochondrial COI, 16S rDNA, and nuclear 18S rDNA, 28S rDNA gene fragments was performed using the following primer sets: LCO1490/HCO709 for COI (Blank et al. 2008), 16Sar/16Sbr for 16S rDNA (Kessing et al. 1989), 18E/18B for 18S rDNA (Mincks et al. 2009), and C1/C2 for 28S rDNA (Le et al. 1993). Molecular analyses were performed using the partial sequences aligned by Geneious v. 8.1.9 (Biomatters Auckland New Zealand). A maximum-likelihood (ML) tree was constructed based on the concatenated partial sequences of three gene regions (16S rDNA (313 bp), 18S rDNA (1,725 bp), and 28S rDNA (305 bp)) using IQ-TREE with the TNe+I+G4 model with 1000 replicates (Kalyaanamoorthy et al. 2017; Hoang et al. 2018). The sequence of *Boccardiaproboscidea* Hartman, 1940 was used as an outgroup taxon (Radashevsky et al. 2014). The DNA sequences determined in the present study were registered in GenBank.

## ﻿Results

### ﻿Systematics

**Family Spionidae Grube, 1850**

#### Genus *Rhynchospio* Hartman, 1936

##### ﻿ Rhynchospio aciliata sp. nov.

Taxon classificationAnimaliaSpionidaSpionidae

FC8BB259-6E20-5499-BE65-08815F39BD41

http://zoobank.org/1F611CE8-58B5-48B3-9462-DF48AEE0FF0A

Figs 2 , 3 , 4 , 5

###### Type locality.

Korea Strait, Korea: Jeollanam-do, Wando-gun, Soan-myeon, Gahak-ri, Soan Island, 34°9'56.1"N, 126°39'29.8"E, intertidal sandflats.

**Figure 2.**
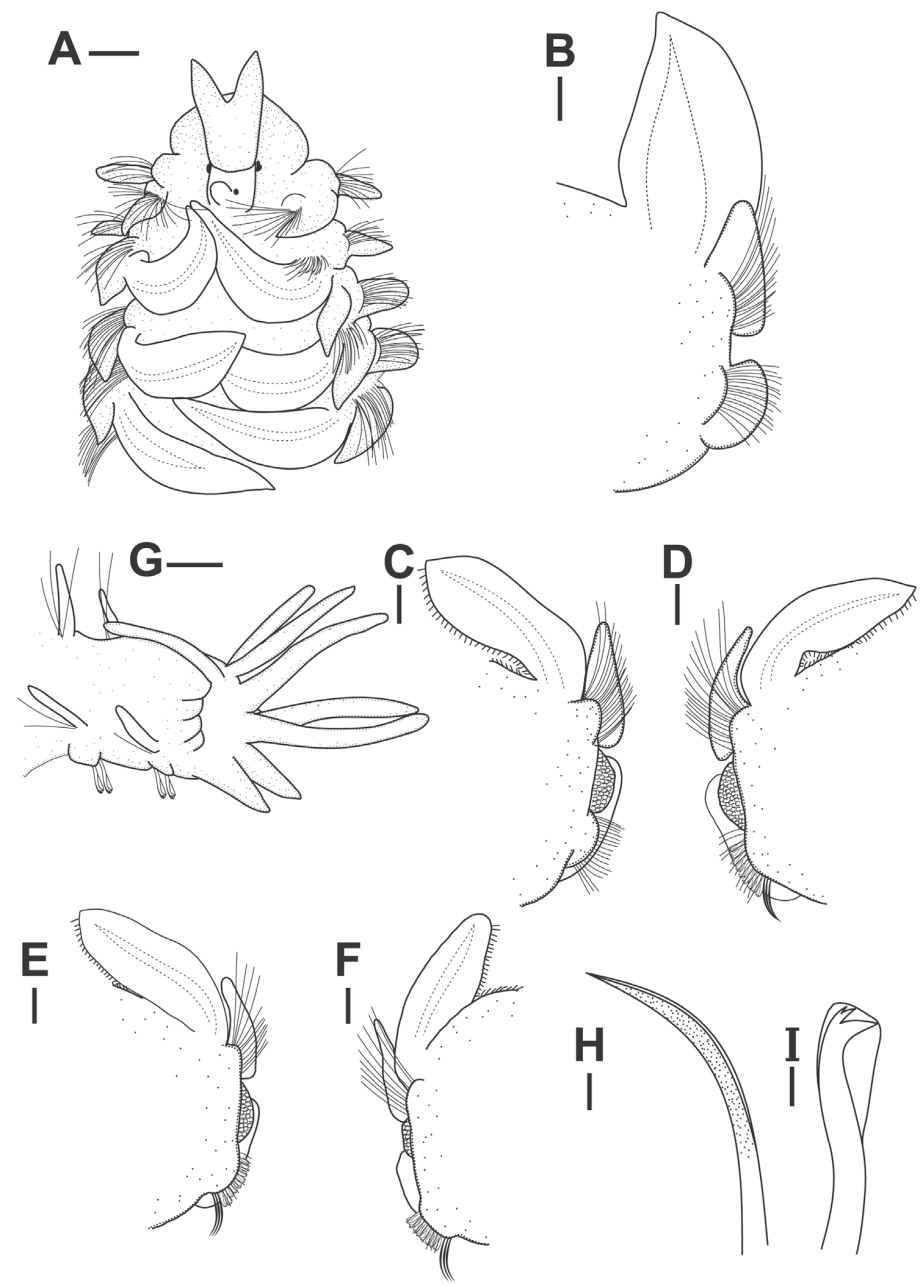
*Rhynchospioaciliata* sp. nov. **A, G** holotype (NIBRIV0000893855) **B–F, H, I** paratype (NIBRIV0000893854) **A** anterior end, dorsal view **B–F** parapodium from chaetiger 5, 19, 23, 35, 49, all anterior views **G** posterior end, left four dorsal cirri removed, lateral view **H** ventral sabre chaeta of chaetiger 31 **I** neuropodial hooded hook of chaetiger 31. Scale bars: 0.2 mm (**A–F**); 0.1 mm (**G**); 20 μm (**H, I**).

###### Material examined.

**Figure 3.**
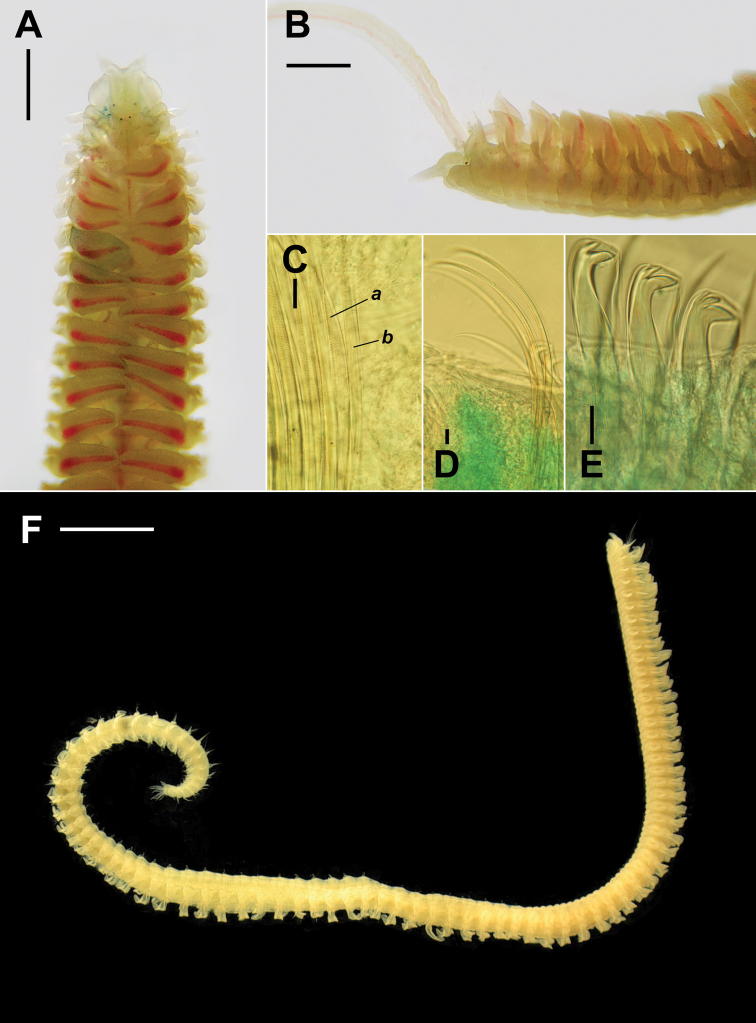
*Rhynchospioaciliata* sp. nov. **A–E** paratype (NIBRIV0000893853) **F** holotype (NIBRIV0000893855) **A, B** live specimen in seawater, dorsal view (**A**) and lateral view of anterior end (**B**) **C** neuropodial chaetae in chaetiger 35, anterior-row chaeta with heavy granulations (a) and posterior-row chaeta without granulation (b) **D** ventral sabre chaetae in chaetiger 35 **E** neuropodial hooded hooks in chaetiger 31 **F** whole body, removed palps, fixed in formalin. Scale bars: 0.5 mm (**A, B**); 20 μm (**C–E**); 2 mm (**F**).

***Holotype***: complete specimen (NIBRIV0000893855) (Fig. 3F), fixed in formalin. ***Paratypes***: five complete (NIBRIV0000893849–53), 12 complete, 23 anterior fragments, 7 middle fragments, 9 posterior fragments (NIBRIV0000893854) fixed in formalin; 2 complete (NIBRIV0000893847–8), 1 anterior fragment (NIBRIV0000893846), 95% ethanol. All examined specimens were collected from the type locality, 25 May 2021.

###### Diagnosis.

Prostomium with 2 conical pointed frontolateral horns, extending posteriorly to anterior margin of chaetiger 1, with papilliform elevation on posterior part. Metameric nuchal organs brownish and oval, ciliary bands double-paired from chaetiger 1 to chaetigers 42–49. First notopodial postchaetal lamellae large and lanceolate. Anterior branchiae conspicuously large and foliaceous. Tridentate hooded hooks appearing from chaetigers 18–24, numbering 9 or 10 per fascicle. Transverse ciliated bands, ciliation on the inner branchiae, and intersegmental transverse cilia absent in anteriormost chaetigers. Sperm from chaetiger 12 to chaetigers 34–43. Oocytes from chaetigers 35–44 onwards, up to approximately 120 μm in diameter. Pygidium with 1 pair of stout, conical ventral cirri, and usually 4–6 pairs of thin, long dorsolateral cirri.

###### Description.

Holotype specimen complete with 85 chaetigers, approximately 0.9 mm wide and 7.0 mm long. Paratypes with 79–93 chaetigers, 0.8–1.1 mm wide, and 5.5–10.7 mm long. Yellowish-white in both live and formalin-fixed specimens (Fig. 3A, B, F).

**Figure 4.**
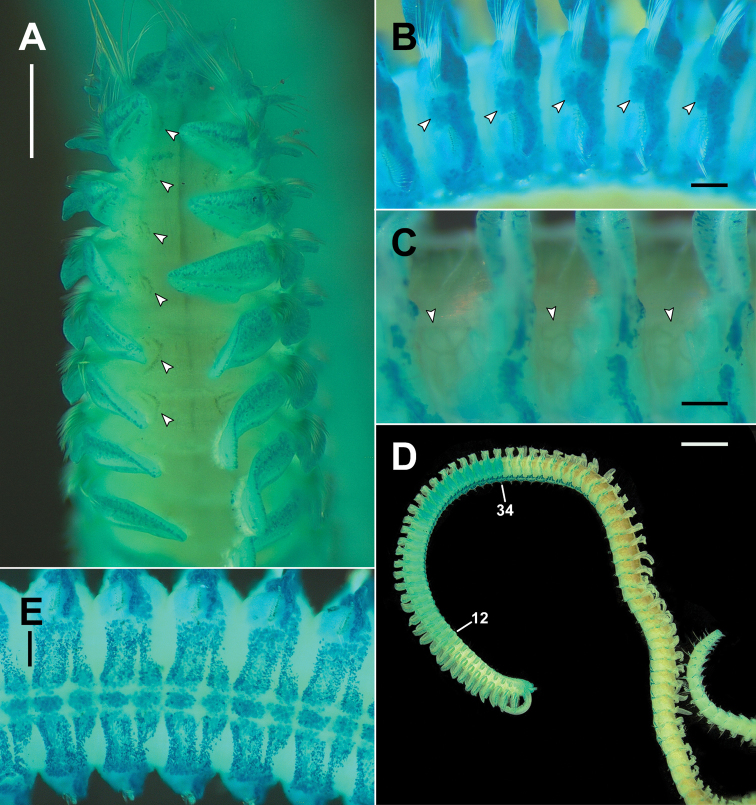
*Rhynchospioaciliata* sp. nov. **A–C** paratype (NIBRIV0000893852) **D, E** paratype (NIBRIV0000893851) fixed in formalin, with a solution of methyl green in distilled water **A** dorsal view of anterior end, nuchal organs (arrowheads) **B** lateral view of chaetigers 19–23, intersegmental lateral pouches (arrowheads) **C** lateral view of chaetigers 66–69, oocytes (arrowheads) **D** lateral view of whole body, at least one week after staining **E** ventral view of chaetigers 25–30. Scale bars: 0.5 mm (**A**); 0.2 mm (**B, C, E**); 2 mm (**D**).

**Figure 5.**
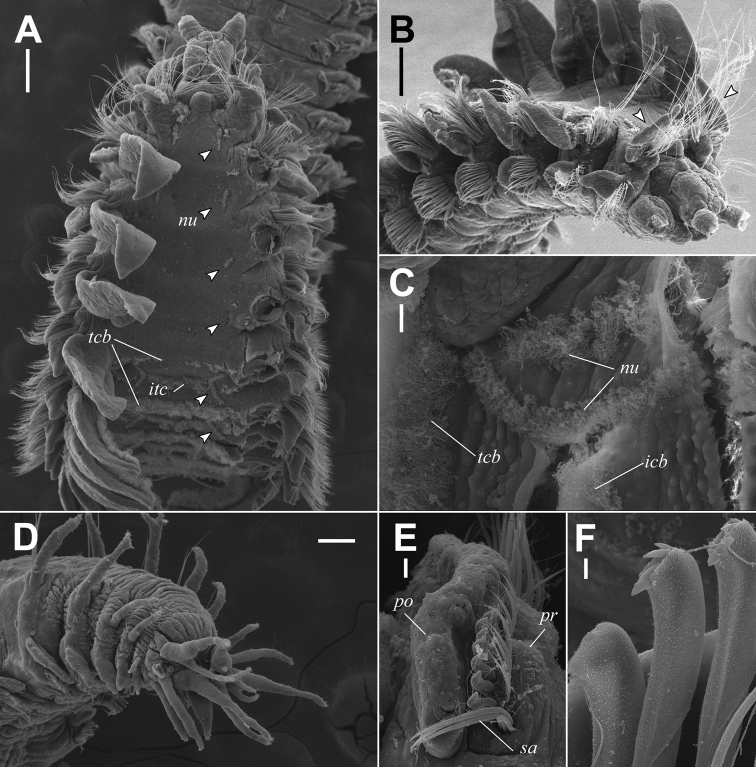
Scanning electron microscopy observation of *Rhynchospioaciliata* sp. nov., paratype (NIBRIV0000893850) **A** dorsal view of anterior end with right branchiae removed, nuchal organs (arrowheads) **B** lateral view of anterior end, first notopodial postchaetal lamellae (arrowheads) **C** double-paired ciliary bands on right side of chaetiger 41 **D** pygidium, lateral view **E** neuropodium of chaetiger 22, ventrolateral view **F** neuropodial hooded hooks from chaetiger 45, lateral view. *nu*: metameric nuchal organs, *tcb*: transverse ciliated bands, *itc*: intersegmental transverse cilia, *pr*: prechaetal lobe, *po*: postchaetal lobe, *sa*: ventral sabre chaetae. Scale bars: 0.2 mm (**A, B**); 20 μm (**C, F**); 0.1 mm (**E**); 4 μm (**G**).

Prostomium with 2 conical pointed frontolateral horns; transverse depression between anterior and middle part of prostomium (Fig. 5A); 2 pairs of black eyes arranged in a trapezoid, anterior pair crescent-shaped, more widely separated than posterior pair, and posterior pair round or slightly crescent-shaped; caruncle low and indistinct, extending posteriorly to anterior margin of chaetiger 1; conspicuous papilliform elevation on posterior part of prostomium; occipital antenna absent (Figs 2A, 5A). Peristomium moderately developed, not forming lateral wings, partially encompassing prostomium posteriorly (Fig. 5B). Palp reaching back to chaetigers 4–7, with longitudinal groove lined with fine cilia. Nuchal organs metameric, brownish, oval (arrowheads in Fig. 4A), ciliary bands double-paired (Fig. 5C) from chaetiger 1 to chaetigers 42–49 (46 in holotype); first four pairs slightly curved or almost straight; from chaetiger 5, nuchal organs conspicuously curved inward (Fig. 5A, C).

**Figure 6.**
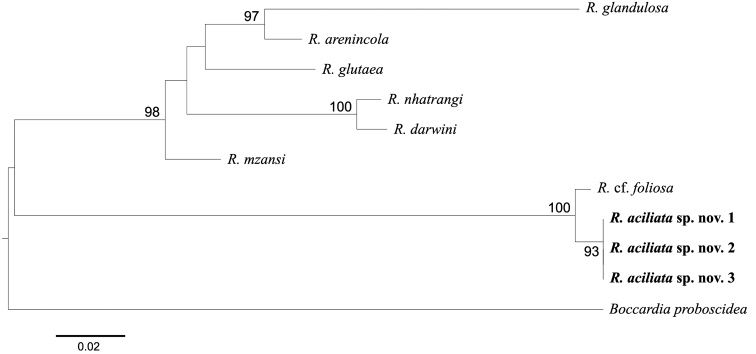
Maximum-likelihood (ML) tree for 2343 bp inferred from combined partial mitochondrial 16S rDNA (313 bp), nuclear 18S ribosomal DNA (rDNA) (1,725 bp), 28S rDNA (305 bp) from nine spionid polychaetes. Numbers above the branch indicate ML bootstrap values from 1000 replications. The sequence of *Boccardiaproboscidea* was used for outgroup rooting.

Chaetiger 1 well developed, with large, lanceolate notopodial postchaetal lamellae and conical neuropodial postchaetal lamellae (Fig. 5B); notochaetae present. Branchiae from chaetiger 2 present almost throughout body, absent on last 5–9 chaetigers (Fig. 6D); anterior branchiae large, broad, and foliaceous, gradually becoming narrower and smallest in posterior chaetigers (Fig. 2B–F); branchiae separated from notopodial postchaetal lamellae; branchiae with distinct ciliation at inner margin present from chaetiger 6 (Figs 4A, 5A). Notopodial postchaetal lamellae lanceolate to subtriangular, largest in anterior chaetigers (Fig. 2B), gradually becoming small with pointed tips in posterior chaetigers (Fig. 2C–F). Neuropodial postchaetal lamellae conical with rounded tips in first 2 chaetigers, becoming broadly rounded until about chaetiger 15, and becoming broad, low subrectangular posteriorly (Fig. 2B–F). Prechaetal lobes low and rounded in both rami (Fig. 5E).

Chaetae in notopodia all capillaries with sheaths; arranged in 2 indistinct rows in anterior chaetigers, more posteriorly arranged in a bundle; 8–12 capillaries very long, non-granulated capillaries in superiormost position at first 2 chaetigers (Fig. 2A); anterior-row capillaries slightly with heavy granulations and posterior-row capillaries longer, non-granulated in anterior chaetigers; granulations of anterior-row capillaries becoming faint posteriorly and completely disappear from chaetigers 40–50. Chaetae in neuropodia with sheathed capillaries and hooded hooks as well as sabre chaetae in inferior position, arranged in 2 rows (Fig. 5E); chaetae in anterior row shorter and stouter, heavily granulated and capillaries in posterior row rather thin, non-granulated in anterior chaetigers (Fig. 3C); 9 or 10 tridentate hooded hooks replacing posterior row of neurochaetae from chaetigers 18–24 (usually 19–21, 20 in holotype), covered with minute bristles (Fig. 5F); hooks with 2 small, upper teeth arranged in line above main fang (Figs 2I, 3E, 5F); 4 or 5 capillaries in inferiormost position usually present 2 or 3 segments before hook-bearing chaetigers; 4 or 5 sabre chaetae heavily granulated, with sheaths, appearing from about hook-bearing chaetigers (Figs 2H, 3D, 5E).

Transverse ciliated bands and intersegmental transverse cilia present from chaetiger 5 to almost throughout body (Fig. 5A), absent on last 6–9 chaetigers (Fig. 6D). Intersegmental lateral pouches positioned in front of superior part of neuropodial postchaetal lamellae, small in the beginning, appearing from chaetigers 13–15 (Fig. 2C–F). Glandular pouches indiscernible. Sperm from chaetiger 12 to chaetigers 34–43 (35 in holotype). Oocytes subspherical, from chaetigers 35–44 onwards (35 in holotype), up to approximately 120 μm in diameter (Fig. 4C); envelope approximately 5 μm thick, with external honey-combed surface but without vesicles at inner surface; single nucleolus approximately 12 μm in diameter. Embryos not observed in any of the examined specimens.

Pygidium with 1 pair of stout, conical ventral cirri, and usually 4–6 pairs (5 pairs in holotype, 1 specimen (NIBRIV0000893849) with 24 pairs) of thin, long dorsolateral cirri (Fig. 2G); occasionally some dorsolateral cirri bifid (Fig. 5D).

*MGSP*. Prostomium, peristomium, basal part of palps, margins of branchiae, intersegmental lateral pouches (Fig. 4B), notopodial, and neuropodial lobes were intensely stained (Fig. 4C). On the dorsal side, transverse ciliated bands across the dorsum were not distinctly stained (Fig. 4A). On the ventral side, 2 transverse bands per chaetiger were stained intensely (Fig. 4E). For at least 1 week after staining, the prostomium, peristomium, chaetiger 12 to chaetigers 34–43 remained stained (male fertile segments), and the ventral transverse bands in anterior and middle chaetigers conspicuously remained (Fig. 4D).

###### Etymology.

The specific name *aciliata* is a combination of the Latin prefix *a*- and the Latin word *cilia*, meaning “absence of cilia.” This name refers to the absence of ciliation on the dorsum and inner margins of branchiae of the anteriormost chaetigers.

###### Habitat and ecology.

The new species was found in fine sand in the intertidal zone.

###### Distribution.

Soan Island, Korea.

###### Genetics.

The partial mitochondrial COI, 16S rDNA, nuclear 18S rDNA, and 28S rDNA sequences from three specimens of *R.aciliata* sp. nov. were determined. The GenBank accession numbers and sequence lengths of the species were as follows: ON206852–4 for COI (687 bp), ON206000–2 for 16S rDNA (517 bp), ON206003–5 for 18S rDNA (1,778 bp), and ON206006–8 for 356 bp (28S rDNA) (Table 1). The intra-specific genetic distances were 0–0.4% in 16S rDNA, and no variation was detected in the other three gene regions. Based on the available molecular data of *Rhynchospio* from GenBank, the new species is genetically the closest to R.cf.foliosa from Oregon, USA. The genetic distance between the new species and R.cf.foliosa was 6.9% (22/294 bp, KR607514) in 16S rDNA, 0.4% (4/1,723 bp, KR607515) in 18S rDNA, and 0.7% (2/351 bp, KP986490) in 28S rDNA (Table 2). Phylogenetic analyses showed that *R.aciliata* sp. nov. formed a monophyletic clade with R.cf.foliosa from Oregon, USA (Fig. 6). This result implies that their close relationships share several morphological characteristics (see below). Unfortunately, the molecular information of *R.foliosa* from Japan (type locality) is still unknown. Further genetic studies on *R.foliosa* are needed to confirm their genetic relationships.

**Table 1.** GenBank accession numbers of *Rhynchospio* species and outgroup taxon used for phylogenetic analysis.

	Species	GenBank accession number			References
		16S rDNA	18S rDNA	28S rDNA	
1	*R.aciliata* sp. nov.	ON206000–2	ON206003–5	ON206006–8	Present study
2	*R.arenincola*	KJ546328	KJ546291	KJ546232	Radashevsky et al. (2014)
3	R.cf.foliosa	KP986488	KP986489	KP986490	Radashevsky et al. (2016)
4	*R.darwini*	KP986492	KP986493	KP986494	Radashevsky et al. (2016)
5	*R.glandulosa*	KJ546344	KJ546295	KJ546246	Radashevsky et al. (2014)
6	*R.glutaea*	KJ546334	KJ546283	KJ546243	Radashevsky et al. (2014)
7	*R.mzansi*	MF625254	MF625258	MF625262	Simon et al. (2018)
8	*R.nhatrangi*	KJ546342	KJ546299	KJ546250	Radashevsky et al. (2014)
9	*Boccardiaproboscidea*	KJ546323	KJ546254	KJ546204	Radashevsky et al. (2014)

**Table 2.** Genetic distances of four molecular markers (cytochrome c oxidase subunit I, 16S ribosomal DNA [rDNA], 18S rDNA, and 28S rDNA) (uncorrected pairwise distances) within and among the new species and other *Rhynchospio* species.

Species		GenBank accession number	Uncorrected pairwise distances							References
**Cytochrome c oxidase I (624 bp aligned)**			1	2						
1	*R.aciliata* **sp. nov.**	ON206852	identical							Present study
		ON206853								
		ON206854								
2	*R.glutaea*	KM998739	20.2%							Radashevsky et al. (unpublished)
**16S rDNA (294 bp aligned)**			1	2	3	4	5	6	7	
1	*R.aciliata* **sp. nov.**	ON206000	0–0.4%							Present study
		ON206001								
		ON206002								
2	R.cf.foliosa	KR607514	6.9%							Radashevsky et al. (2016)
3	*R.mzansi*	MF625254	18.8%	18.7%						Simon et al. (2018)
4	*R.nhatrangi*	KJ546342	21.3%	21.5%	16.0%					Radashevsky et al. (2014)
5	*R.glutaea*	KJ546332	21.6%	21.5%	12.8%	17.1%				Radashevsky et al. (2014)
6	*R.darwini*	KP986492	22.3%	22.2%	16.7%	10.0%	17.7%			Radashevsky et al. (2016)
7	*R.arenincola*	KJ546331	23.3%	22.8%	18.7%	17.7%	14.2%	21.2%		Radashevsky et al. (2014)
8	*R.glandulosa*	KJ546347	24.4%	24.3%	22.0%	22.0%	21.3%	25.4%	17.1%	Radashevsky et al. (2014)
**18S rDNA (1,723 bp aligned)**			1	2	3	4	5	6	7	
1	*R.aciliata* **sp. nov.**	ON206003	identical							Present study
		ON206004								
		ON206005								
2	R.cf.foliosa	KR607515	0.4%							Radashevsky et al. (2016)
3	*R.arenincola*	KJ546286	11.0%	11.1%						Radashevsky et al. (2014)
4	*R.mzansi*	MF625258	11.2%	11.0%	2.0%					Simon et al. (2018)
5	*R.glutaea*	KJ546281	11.6%	11.6%	2.5%	2.7%				Radashevsky et al. (2014)
6	*R.nhatrangi*	KJ546299	12.7%	12.8%	4.0%	3.9%	4.5%			Radashevsky et al. (2014)
7	*R.darwini*	KP986493	12.8%	12.9%	4.0%	4.0%	4.5%	0.2%		Radashevsky et al. (2016)
8	*R.glandulosa*	KJ546295	13.6%	13.5%	5.6%	6.4%	6.6%	7.7%	7.8%	Radashevsky et al. (2014)
**28S rDNA (301 bp aligned)**			1	2	3	4	5	6	7	
1	*R.aciliata* **sp. nov.**	ON206006	identical							Present study
		ON206007								
		ON206008								
2	R.cf.foliosa	KP986490	0.7%							Radashevsky et al. (2016)
3	*R.mzansi*	MF625262	13.0%	13.0%						Simon et al. (2018)
4	*R.arenincola*	KJ546232	14.3%	14.3%	3.4%					Radashevsky et al. (2014)
5	*R.glutaea*	KJ546243	14.7%	14.7%	4.1%	4.4%				Radashevsky et al. (2014)
6	*R.nhatrangi*	KJ546250	14.7%	14.7%	4.1%	4.4%	5.8%			Radashevsky et al. (2014)
7	*R.darwini*	KP986493	14.7%	14.7%	4.1%	4.7%	5.8%	0.3%		Radashevsky et al. (2016)
8	*R.glandulosa*	KJ546246	18.0%	18.0%	6.8%	8.5%	8.2%	8.5%	8.8%	Radashevsky et al. (2014)

## ﻿Discussion

Seven *Rhynchospio* species are known to be simultaneous hermaphrodites, three of which brood their larvae on the parent’s dorsum (Radashevsky and Choi 2021). *Rhynchospioaciliata* sp. nov. examined in this study is a simultaneous hermaphrodite producing spermatozoa and oocytes in the anterior and posterior chaetigers respectively, but no brooding embryo was observed in any of the materials collected in May 2021. This finding is quite different from that of *R.glandulosa* specimens found with larvae on the dorsum collected in May 2013 and May to June 2016–2018. It seems likely that the brooding period is species-specific. The reproductive biology and sperm morphology of this new species is unknown.

The new species is unique in terms of the lack of ciliation in the anteriormost chaetigers. Among the known *Rhynchospio* species, *R.foliosa* from Japan has an elevation on the prostomium and conspicuously large, foliaceous anterior branchiae (Imajima 1991; see a key to the species by Radashevsky et al. 2014). We examined the non-type material of *R.foliosa* (NSMT-Pol 104819, collected by Imajima in 1991, from Hokkaido (43°00.9'N, 144°49.6'E) near the type locality) and it agreed well with the original description. *Rhynchospioaciliata* sp. nov. is morphologically very similar to *R.foliosa* in having large, foliaceous branchiae and the presence of intersegmental lateral pouches (described as “membranous ridge” by Imajima (1991)). However, the new species clearly differs from the Japanese species (characteristics present in parentheses) by large, lanceolate notopodial postchaetal lamellae of chaetiger 1 (small and similar to those of neuropodia), papilliform elevation in the posterior part (caruncle anteriorly elevated above the prostomium), ciliation absent in the anteriormost chaetigers (present), neuropodial hooks numbering 9 or 10 per fascicle (8 per fascicle), and pygidium with a pair of ventral cirri and elongated dorsolateral cirri (numerous foliaceous lobes) (Imajima 1991). The new species is also similar to R.cf.foliosa from USA in sharing the large and foliaceous branchiae, oocytes with a thick honey-combed envelope, and a pygidium with paired ventral cirri and numerous pairs of long, slender cirri (Radashevsky et al. 2016). The new species, however, differs from the latter species by the metameric nuchal organs present in the first 42–49 segments (about first 25 segments in R.cf.foliosa), ventral sabre chaetae present from chaetigers 18–24 (25–30), sperm present from chaetiger 12 (chaetiger 13), and a pygidium without pigmentation on both live and fixed specimens (ventral cirri of pygidium with dark pigmentation) (Radashevsky et al. 2016). Within Korean waters, a member of the *R.glutaea* complex *sensu*Radashevsky et al. (2014), *R.glandulosa*, is known to inhabit the intertidal zone and shallow waters (Radashevsky and Choi 2021). The new species can be easily distinguished from *R.glandulosa* by the presence of a papilliform elevation on the caruncle (absent in *R.glandulosa*), conspicuously large and foliaceous branchiae (moderate size and elongate), neuropodial hooded hooks appearing from chaetigers 18–24 (12–17), sperm present up to 32 segments (up to 4 segments), and pygidium with up to 15 pairs (up to 4 pairs).

Simon et al. (2018) illustrated the MGSP of *R.mzansi* from South Africa for the first time in this genus. The new species and *R.mzansi* have similar staining patterns in having 2 transverse bands ventrally but differ in the dorsal side of the body: indistinctly stained in the new species and distinctly stained (especially anterior chaetigers) in *R.mzansi* (Simon et al. 2018). After at least 1 week after staining, the region of the chaetigers with sperm (male fertile chaetigers) remained conspicuously stained (in all examined specimens) but was not as prominent as that of the ventral transverse bands. These findings support the finding of Simon et al. (2018) that the MGSP is a reliable character for species identification in the genus *Rhynchospio*.

Phylogenetic analysis based on the sequences of three gene regions (16S rDNA, 18S rDNA, and 28S rDNA) showed that two monophyletic clades: *R.aciliata* sp. nov. and R.cf.foliosa in one clade, and all other known species in a second clade. The most conspicuous morphological features separating the two groups seem to be the branchial morphology (large and foliaceous vs elongated and normal in size) and the arrangement of male segments (much more than 12 segments vs not more than 12 segments).

The gene sequences obtained in this study along with morphological information, including MGSP and SEM observations, will be useful for further taxonomic or phylogenetic studies of genus *Rhynchospio*.

## Supplementary Material

XML Treatment for Rhynchospio aciliata

## References

[B1] Abe H Sato-Okoshi W (2021) Molecular identification and larval morphology of spionid polychaetes (Annelida, Spionidae) from northeastern Japan. ZooKeys 1015: 1–86. 10.3897/zookeys.1015.54387 33613041 PMC7878468

[B2] Blake JA (1996) Family Spionidae Grube, 1850. Including a review of the genera and species from California and a revision of the genus *Polydora* Bosc, 1802. In: Blake JA Hilbig B Scott PH (Eds) Taxonomic Atlas of the Benthic Fauna of the Santa Maria Basin and Western Santa Barbara Channel.Volume 6 – The Annelida Part 3, Polychaeta: Orbiniidae to Cossuridae. Santa Barbara Museum of Natural History Press, Santa Barbara, 81–223.

[B3] Blake JA Kudenov JD (1978) The Spionidae (Polychaeta) from southeastern Australia and adjacent areas with a revision of the genera. Memoirs of the National Museum of Victoria 39: 171–280. 10.24199/j.mmv.1978.39.11

[B4] Blank M Laine A Jürss K Bastrop R (2008) Molecular identification key based on PCR/RFLP for three polychaete sibling species of the genus *Marenzelleria*, and the species’ current distribution in the Baltic Sea. Helgoland Marine Research 62(2): 129–141. 10.1007/s10152-007-0081-8

[B5] Chlebovitsch VV (1959) Species of Polychaeta worms from the Kurile Islands, which are new or recorded for the first time in the USSR fauna. Zoologicheskij Zhurnal 38: 167–181.

[B6] Fauchald K (1977) The polychaete worms. Definitions and keys to the orders, families and genera. Natural History Museum of Los Angeles County. Science Series 28: 1–190.

[B7] Hoang DT Chernomor O Haeseler A Minh BQ Vinh LS (2018) UFBoot2: Improving the ultrafast bootstrap approximation. Molecular Biology and Evolution 35(2): 518–522. 10.1093/molbev/msx281 29077904 PMC5850222

[B8] Imajima M (1991) Spionidae (Annelida, Polychaeta) from Japan. VI. The genera *Malacoceros* and *Rhynchospio*. Bulletin of the National Science Museum, Tokyo, Series A (Zoology) 17: 5–17.

[B9] Kalyaanamoorthy S Minh BQ Wong TKF von Haeseler A Jermiin LS (2017) ModelFinder: Fast model selection for accurate phylogenetic estimates. Nature Methods 14(6): 587–589. 10.1038/nmeth.4285 28481363 PMC5453245

[B10] Kessing B Croom H Martin A McIntosh C Owen McMillian W Palumbi S (1989) The Simple Fool’s Guide to PCR. Department of Zoology, University of Hawaii, Honolulu, 47 pp.

[B11] Le HLV Lecointre G Perasso R (1993) A 28S rRNA-based phylogeny of the gnathostomes: First steps in the analysis of conflict and congruence with morphologically based cladograms. Molecular Phylogenetics and Evolution 2(1): 31–51. 10.1006/mpev.1993.1005 8081546

[B12] Mincks SL Dyal PL Paterson GLJ Smith CR Glover AG (2009) A new species of *Aurospio* (Polychaeta, Spionidae) from the Antarctic shelf, with analysis of its ecology, reproductive biology and evolutionary history. Marine Ecology (Berlin) 30(2): 181–197. 10.1111/j.1439-0485.2008.00265.x

[B13] Meißner K (2005) Revision of the genus *Spiophanes* (Polychaeta: Spionidae); with new synonymies, new records and descriptions of new species. Zoosystematics and Evolution 81(1): 3–66. 10.1002/mmnz.200310001

[B14] Radashevsky VI (2007) Morphology and biology of a new *Rhynchospio* species (Polychaeta: Spionidae) from the South China Sea, Vietnam, with the review of *Rhynchospio* taxa. Journal of Natural History 41(17): 985–997. 10.1080/00222930701376717

[B15] Radashevsky VI Choi JW (2021) Morphology and reproductive biology of a new hermaphroditic *Rhynchospio* (Annelida: Spionidae) species brooding larvae on the parent’s dorsum. Marine Biodiversity 51(4): 1–15. 10.1007/s12526-021-01197-6

[B16] Radashevsky VI Neretina TV Pankova VV Tzetlin AB Choi JW (2014) Molecular identity, morphology and taxonomy of the *Rhynchospioglutaea* complex with a key to *Rhynchospio* species (Annelida, Spionidae). Systematics and Biodiversity 12(4): 424–433. 10.1080/14772000.2014.941039

[B17] Radashevsky VI Malyar VV Pankova VV Nuzhdin SV (2016) Molecular analysis of six *Rhynchospio* Hartman, 1936 species (Annelida: Spionidae) with comments on the evolution of brooding within the group. Zootaxa 4127(3): 579–590. 10.11646/zootaxa.4127.3.10 27395642

[B18] Simon CA Williams LG Henninger T (2018) A new species of *Rhynchospio* (Annelida: Spionidae) in South Africa. Marine Biodiversity 49(2): 663–672. 10.1007/s12526-017-0842-9

